# Neurologische Komplikationen der Hepatitis-C-Infektion

**DOI:** 10.1007/s00115-020-00999-6

**Published:** 2020-10-01

**Authors:** Felix Kleefeld, Gabriele Arendt, Eva Neuen-Jacob, Matthias Maschke, Ingo Husstedt, Mark Obermann, Holger Schmidt, Katrin Hahn, Deutsche Gesellschaft für Neuro-AIDS und Neuro-Infektiologie (DGNANI)

**Affiliations:** 1 Klinik für Neurologie, Universitätsmedizin Charité, Charitéplatz 1, 10117 Berlin, Deutschland; 2 Neurologie, Neuro-Centrum Düsseldorf, Hohenzollernstr. 5, 40211 Düsseldorf, Deutschland; 3 Institut für Neuropathologie, Universitätsklinikum Düsseldorf, Moorenstraße 5, 40225 Düsseldorf, Deutschland; 4 Klinik für Neurologie, Krankenhaus der Barmherzigen Brüder, Nordallee 1, 54292 Trier, Deutschland; 5 Praxis an der Klinik Maria Frieden, Am Krankenhaus 1, 48291 Telgte/Münster, Deutschland; 6 Klinik für Neurologie, Asklepios Kliniken Schildautal, Karl-Herold-Str. 1, 38723 Seesen, Deutschland; 7 Klinik für Neurologie, Elbe-Kliniken Stade, Bremervörder Str. 111, 21682 Stade, Deutschland

**Keywords:** Hepatitis C, Polyneuropathie, Kognitive Defizite, Kryoglobulinämie, Small-fiber-Neuropathie, Hepatitis C, Polyneuropathy, Cognitive deficits, Cryoglobulinemia, Small fiber neuropathy

## Abstract

Die chronische Hepatitis-C-Virus(HCV)-Infektion ist eine hochprävalente Systemerkrankung, die verschiedene neurologische Komplikationen verursachen kann. Es lassen sich HCV-assoziierte Symptome im zentralen und peripheren Nervensystem sowie der Muskulatur unterscheiden. Wichtige Pathomechanismen sind die HCV-assoziierte Autoimmunität (z. B. gemischte Kryoglobulinämie mit Polyneuropathie) und direkte Neurotoxizität (z. B. bei HCV-assoziierten kognitiven Defiziten). Die häufigsten neurologischen Komplikationen sind distal-symmetrische Polyneuropathien, Small-fiber-Neuropathien und kognitive Defizite. Die HCV-Infektion stellt außerdem einen Risikofaktor für ischämische und hämorrhagische Schlaganfälle sowie den Morbus Parkinson dar. Die frühe Identifikation und antivirale Behandlung HCV-positiver Patienten steht im Zentrum der Behandlung. Durch neue antivirale Therapien können >90 % der Patienten dauerhaft von der HCV-Infektion geheilt werden.

## Abstract (en)

Chronic hepatitis C virus (HCV) infection is a highly prevalent systemic disease, which can cause a variety of neurological complications. The HCV-associated symptoms can be differentiated into central and peripheral nervous systems as well as the musculature. Important pathomechanisms are HCV-associated autoimmunity (e.g. mixed cryoglobulinemia with polyneuropathy) and direct neurotoxic effects of the virus (e.g. HCV-associated cognitive deficits). Distal symmetric polyneuropathies, small fiber neuropathies and cognitive deficits are the most prevalent neurological manifestations. Furthermore, HCV infection is a risk factor for ischemic and hemorrhagic stroke as well as Parkinson’s disease. As HCV infection has become a permanently curable disease in >90% of patients, early identification and antiviral treatment of HCV positive patients is of utmost importance.

## Hintergrund

Geschätzt 180 Mio. Menschen sind weltweit mit dem Hepatitis-C-Virus (HCV) infiziert. In Deutschland lassen sich bei etwa 0,5 % der Bevölkerung Antikörper gegen HCV nachweisen [1]. Die Hepatitis C ist somit eine hochprävalente und im klinischen Alltag häufig anzutreffende Infektionskrankheit. Seit 2014 lässt sich die chronische HCV-Infektion durch den Einsatz direkt wirkender antiviraler Substanzen („direct acting antivirals“, DAAs) effektiv und nebenwirkungsarm behandeln und bei >90 % der Patienten dauerhaft heilen [2]. Die heute verfügbaren DAAs umfassen Proteaseinhibitoren, NS5A- sowie NS5B-Inhibitoren und greifen somit gezielt in den Replikationszyklus des Virus ein.

**Figure 1:**
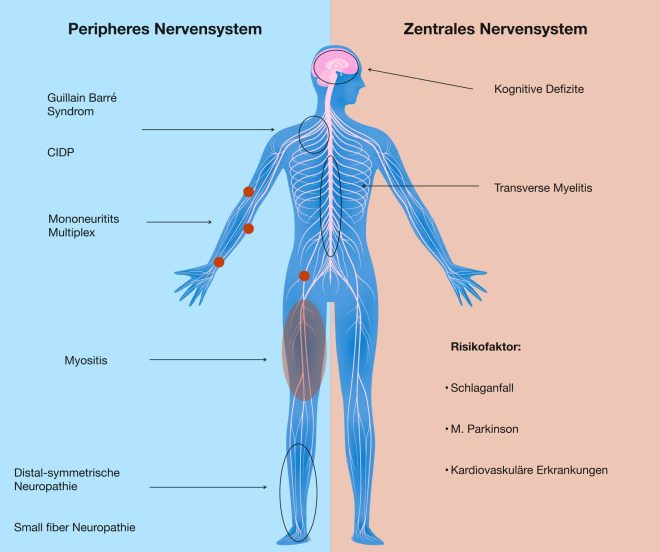

Es ist gut belegt, dass HCV nicht nur hepatotrop, sondern auch lymphotrop wirksam ist [3, 4]. Dies bedingt, dass das Virus neben einer chronisch-aktiven Hepatitis auch eine Vielzahl extrahepatischer Manifestationen verursachen kann. Einige Autoren verwenden daher den Begriff „Hepatitis-C-Syndrom“ [5], um das Spektrum extrahepatischer spezifischer Organmanifestationen, systemischer Autoimmunerkrankungen und Tumoren im Kontext der Infektion zu charakterisieren. Extrahepatische Manifestationen sind häufig Ausdruck einer B‑Zell-Proliferation mit Produktion mono- oder polyklonaler Antikörper. Damit assoziiert ist beispielsweise das Auftreten einer Kryoglobulinämie [6, 7]. Kryoglobuline lassen sich entsprechend der Brouet-Klassifikation [8] in 3 Typen einteilen; wobei sich der II. und III. Typ, welche auch als gemischte Kryoglobulinämie bezeichnet werden, häufig bei Hepatitis-C-Infektionen nachweisen lassen [9, 10]. Das Spektrum der Antikörper umfasst darüber hinaus sowohl antinukleäre Antikörper, Anti SS-A/Anti-SS‑B, ANCA als auch GM1-Gangliosid-Antikörper [11]. Der Phänotyp des HCV-Syndroms scheint multifaktoriell bedingt zu sein, einschließlich genetischer und geografischer Faktoren, sodass sich regionale Unterschiede in der Manifestation ergeben können [6]. Aus diesem Grund ist die Erkrankung klinisch nicht nur für Internisten relevant. Für Neurologen und Psychiater von Bedeutung sind die Manifestationen im zentralen und peripheren Nervensystem sowie der Muskulatur. Die HCV-Infektion wird darüber hinaus mittlerweile als eigenständiger Risikofaktor für verschiedene neurologische (z. B. Demenz, Morbus Parkinson), kardiovaskuläre (z. B. Atherosklerose, Schlaganfall) und metabolische (Diabetes mellitus) Erkrankungen angesehen [12]. In dieser Übersichtsarbeit geben wir einen Überblick über mögliche neurologische Komplikationen der HCV-Infektion (Abb. 1).

## Peripheres Nervensystem und Muskulatur

Das periphere Nervensystem (PNS) ist bei HCV-infizierten Patienten insbesondere durch Polyneuropathien betroffen. Das Spektrum möglicher Erkrankungen beinhaltet distal-symmetrische, häufig schmerzhafte axonale Polyneuropathien (PNP), Small-fiber-Neuropathien (SFN) und seltener auch akute oder chronische Polyradikuloneuropathien wie das Guillain-Barré-Syndrom (GBS) oder die chronische inflammatorische demyelinisierende Polyradikuloneuropathie (CIDP). Auch asymmetrische Neuropathien wie die Mononeuritis multiplex sind beschrieben. Es lassen sich damit chronische (PNP, SFN) von akut-subakut auftretenden (CIDP, GBS) und axonale von demyelinisierenden Neuropathien im Kontext der HCV-Infektion unterscheiden.

Angaben zur Prävalenz Hepatitis-C-assoziierter Neuropathien finden sich in der Literatur kaum. Eine prospektive französische Arbeit mit 321 Patienten beschreibt in 9 % das Vorliegen einer sensiblen Polyneuropathie [13]. Mehrheitlich präsentieren sich Patienten mit einer symmetrischen sensiblen oder sensomotorischen Polyneuropathie und deutlich seltener mit einer Mononeuropathie oder Mononeuropathia multiplex [14, 15].

Die Pathogenese der HCV-assoziierten Neuropathien ist nur unvollständig verstanden. Neben vaskulitischen Veränderungen der Vasa nervosum mit Komplementaktivierung und sekundärer Ischämie werden auch direkte virusabhängige Mechanismen mit sekundärer Inflammation diskutiert [15–17]. HCV-RNA ließ sich in epineuralen Zellen nachweisen [17, 18].

Unabhängig vom Auftreten von KryoglobuIinen finden sich in Suralisbiopsien HCV-positiver Patienten mit PNP gehäuft perivaskuläre inflammatorische Infiltrate mit mononukleären Zellen [19], was die Immunpathogenese der Neuropathien unterstreicht. Neben der Präsenz von Kryoglobulinen vom Typ II und III (gemischte Kryoglobulinämie) ist insbesondere die Erkrankungsdauer ein Risikofaktor für das Auftreten von PNP bei diesen Patienten [10, 20]. Der HCV-Genotyp, Schweregrad der Leberfibrose sowie der Kryokrit, also der prozentuale Anteil präzipitierter Kryoglobuline, gelten hingegen nicht als Risikofaktoren für das Auftreten einer PNP [20].

### Distal-symmetrische Polyneuropathien und Small-fiber-Neuropathien

Distal-symmetrische, sensomotorische axonale PNP stellen eine der häufigsten Komplikationen der HCV-Infektion dar und sind mit dem Auftreten von Kryoglobulinen (CG) und einer Vaskulitis assoziiert. Bis zu 86 % der kryoglobulinpositiven Patienten, aber auch bis zu 44 % der kryoglobulinnegativen Patienten sind von einer PNP betroffen [21]. Somit ist das Vorliegen einer HCV-assoziierten Polyneuropathie auch ohne Nachweis von Kryoglobulinen möglich. Klinisch zeigt sich bei den betroffenen Patienten eine häufig schmerzhafte, vorwiegend sensible und distal-symmetrische Polyneuropathie, die in der Regel einen schleichenden bzw. chronischen Verlauf aufweist. Es bestehen Hinweise, dass bei kryoglobulinnegativen Patienten häufiger auch asymmetrische Neuropathien vom Multiplex-Typ auftreten [10]. Eine Hirnnervenbeteiligung ist bei allen Formen von HCV-assoziierter PNP beschrieben [10].

Neben klassischen Polyneuropathien leiden HCV-positive Patienten auch unter teils isoliert auftretenden Small-fiber-Neuropathien. Im Gegensatz zur klassischen (sog. Large-fiber‑)PNP bestehen bei reinen SFN keine Atrophien, Paresen und keine Areflexie. Patienten mit SFN können sich mit isolierten, schmerzhaften Parästhesien, Temperaturempfindungsstörungen oder autonomen Funktionsstörungen präsentieren. Diese initial oft schwer zuzuordnenden Symptome werden erfahrungsgemäß häufig als Schmerzsyndrome, Fibromyalgie oder Somatisierungsstörung fehldiagnostiziert. Die klinisch-neurologische Untersuchung erbringt ebenso wie die Neurographie bei diesen Patienten in der Regel unauffällige Befunde. Die Diagnose SFN lässt sich mit verschiedenen Methoden diagnostizieren. Je nach Verfügbarkeit kann z. B. eine histomorphologische Beurteilung der intraepidermale Nervenfaserdichte in einer Hautstanzbiopsie erfolgen oder eine funktionelle Charakterisierung z. B. mittels quantitativer sensorischer Testung. Bei Diagnose einer SFN sollten daher unbedingt auch Infektionserkrankungen wie die HCV- oder die häufig auch komorbide vorliegende HIV-Infektion in die differenzialdiagnostischen Überlegungen mit einbezogen werden.

### Therapie

Bei der Therapie der HCV-assoziierten Polyneuropathien ist zwischen der symptomorientierten Basistherapie, im Falle einer kryoglobulinassoziierten vaskulitischen Neuropathie einer immunsuppressiven und einer Hepatitis-C-spezifischen antiviralen, kausalen Therapie der Polyneuropathie zu unterscheiden.

Neuropathische Schmerzen können u. a. mit Antikonvulsiva, Antidepressiva, Opiaten oder topischen Therapien (z. B. Capsaicin-Pflaster) nach herkömmlichen symptomatischen Prinzipien behandelt werden. Aus unserer Erfahrung hat sich bei diesen Patienten aber z. B. Duloxetin gut wirksam gezeigt. Kontrollierte Studien fehlen. In einer offenen Studie mit Patienten mit schmerzhafter Hepatitis-C-assoziierter kryoglobulinämischer Neuropathie zeigte sich ein positiver Effekt von Oxcarbazepin in der Schmerzkontrolle [22].

Bei Patienten mit kryoglobulinassoziierter vaskulitischer Neuropathie muss in Abhängigkeit von der Akuität der Erkrankung der additive Einsatz immunsuppressiver Substanzen wie Steroide, Cyclosporin bis hin zur Plasmapherese diskutiert werden [23, 24].

Grundsätzlich sollte aber bei allen Patienten eine kausale Therapie der HCV-Infektion angestrebt werden. Die Auswahl des entsprechenden Therapieregimes sollte dabei in infektiologischer Hand liegen. Daten zur Wirksamkeit der neuen DAA-basierten Therapien auf Polyneuropathien liegen aktuell noch nicht vor. Da interferonbasierte Therapien aber in der Vergangenheit zum Teil deutliche Verbesserungen der Neuropathien erzielten, ist von einer mindestens vergleichbaren Wirksamkeit der DAAs auszugehen [14, 16].

### Demyelinisierende Polyneuropathien/Polyradikulopathien

Das GBS und die CIDP stellen akute bzw. subakut-chronisch auftretende, immunvermittelte Neuropathien dar. Sie wurden sowohl bei der akuten, als auch der chronischen HCV-Infektion beschrieben [25, 26].

Die immunmodulierende Akuttherapie des HCV-assoziierten GBS bzw. der CIDP unterscheidet sich nicht von der der nicht-HCV-assoziierten Varianten. Sie kann die Gabe von Immunglobulinen, Immunsuppressiva oder Plasmapherese beinhalten. Allerdings konnte in der Vergangenheit gezeigt werden, dass eine zusätzliche antivirale Therapie mit Interferon die Prognose möglicherweise zusätzlich verbessert [27, 28]. Die Wirksamkeit der DAAs in diesem Kontext ist bislang nicht untersucht worden.

### Myopathien

Neben dem PNS kann auch die Muskulatur in seltenen Fällen Manifestationsort extrahepatischer Symptome der HCV-Infektion sein. Insbesondere das Auftreten der Polymyositis und der Einschlusskörpermyositis („inclusion body myositis“, IBM) ist mit der chronischen Hepatitis C assoziiert [29]. So waren bei 28 % der Patienten mit IBM in einer Studie HCV-Antikörper nachweisbar [30]. Die Assoziation der IBM zu einer viralen Infektion ist insofern plausibel, als auch die HIV-Infektion einen etablierten Risikofaktor für das Auftreten einer IBM, insbesondere bei jüngeren Patienten, darstellt. In neueren Untersuchungen profitierten HCV-positive Patienten mit IBM allerdings hinsichtlich ihrer neurologischen Symptomatik nicht von einer Viruseradikation, sodass eine therapeutische Konsequenz der HCV-Infektion im Kontext der IBM nicht gesichert ist [31].

- Die Differenzialdiagnose von distal-symmetrischen, schmerzhaften Neuropathien sowie Schwerpunktneuropathien sollte immer die Hepatitis-C-Infektion beinhalten.
- Isolierte Small-fiber-Neuropathien können sich als klinisch blande, schmerzhafte Neuropathien präsentieren. Zur Diagnosestellung ist in unklaren Fällen eine Hautbiopsie notwendig.
- Immunvermittelte Neuropathien und Myopathien stellen seltene Komplikationen der HCV-Infektion dar.
- Neben symptomatischen Therapiemaßnahmen sollte bei allen Patienten mit HCV-assoziierter Neuropathie eine antivirale Therapie der Grunderkrankung angestrebt werden.

## Zentrales Nervensystem

Die HCV-Infektion kann auch im zentralen Nervensystem (ZNS) verschiedene Komplikationen hervorrufen. Diese umfassen Enzephalitiden, Myelitiden, kognitive Defizite sowie Fatigue. Im Rahmen einer fortgeschrittenen Leberfibrose bei chronischer HCV-Infektion kann es außerdem zu einer hepatischen Enzephalopathie (HE) kommen. Diese Komplikation in Form einer metabolischen Enzephalopathie ist allerdings nicht HCV-spezifisch und wird daher an dieser Stelle nicht besprochen. Pathophysiologisch sind im Gegensatz zur metabolischen Genese bei der HE andere Mechanismen für die ZNS-Komplikationen bei der HCV-Infektion relevant. Zum einen wird eine direkte und indirekte Neurotoxizität des Hepatitis-C-Virus im ZNS angenommen. Die aktive Replikation des Virus im ZNS lässt sich auch durch die Virämie im Liquor nachweisen. Daneben spielen in der Pathogenese offensichtlich auch die Folgen einer chronischen Immunaktivierung eine Rolle, wie z. B. Autoimmunität und eine Dysfunktion der Blut-Hirn-Schranke durch Sekretion proinflammatorischer Zytokine wie Interleukin 1ß (IL-1ß; [32]).

### Kognitive Defizite

Die Erstbeschreibung kognitiver Defizite bei HCV-infizierten Patienten ohne strukturelle Leberveränderungen im Jahre 2002 führte zu einer anhaltenden wissenschaftlichen Debatte [33]. Während viele Arbeiten die Existenz kognitiver Defizite bei HCV-infizierten Patienten reproduzieren konnten, stellten einige Autoren mit ihren Ergebnissen die klinische Relevanz der detektierten Defizite infrage [34]. Bei ausführlichen neuropsychologischen Untersuchungen HCV-positiver Patienten zeigten die Patienten allerdings in den meisten Studien reproduzierbare Defizite in bestimmten kognitiven Domänen. Es handelt sich dabei um die Bereiche Lernen und (Arbeits‑)Gedächtnis, Aufmerksamkeit, Konzentration, Exekutivfunktionen und Feinmotorik [35]. Die HCV-Infektion scheint zu einem charakteristischen Muster kognitiver Defizite zu führen, das möglicherweise auf virusbedingte strukturelle Veränderungen und neuroinflammatorische Prozesse im ZNS zurückzuführen ist. Hier besteht klinisch und möglicherweise auch pathophysiologisch eine Parallele zum HIV-assoziierten kognitiven Defizit (HAND), das ein ähnliches neuropsychologisches Muster aufweist. Das HCV-Syndrom kann zu einer erheblichen Einschränkung der Alltagsfunktion und Lebensqualität der Patienten führen. Neben den beschriebenen kognitiven Defiziten leiden viele Patienten zusätzlich auch unter einer Fatigue-Symptomatik und Depressionen [36]. So überrascht es nicht, dass die erfolgreiche Therapie der HCV-Infektion zu einer Senkung der Mortalität, einer Verbesserung der Fatigue-Symptomatik und der Lebensqualität führen kann [36, 37]. Auch eine Verbesserung der Kognition nach erfolgreicher HCV-Therapie ist dokumentiert [35]. Im Zweifel sollten Patienten mit subjektiver kognitiver Verschlechterung bei HCV-Infektion deshalb niedrigschwellig auf kognitive Defizite gescreent und gegebenenfalls neuropsychologisch getestet werden. Eine HCV-Eradikation bietet bei dieser Patientengruppe die seltene Chance einer kausalen Therapie des kognitiven Defizits und sollte deshalb nicht verpasst werden.

### Myelitis

Als Myelitis werden inflammatorische Veränderungen des Rückenmarks bezeichnet, die sowohl die weiße als auch die graue Substanz betreffen können. Man unterscheidet langstreckige inflammatorische Veränderungen von kurzstreckigen Formen der Myelitis (transverse Myelitis). Rezidivierende, transverse Myelitiden können im Kontext einer HCV-Infektion auftreten. In einer Fallserie kam es bei HCV-positiven Patienten mit transverser Myelitis zu 2 bis 5 Rezidiven [38]. Andere chronisch-entzündliche ZNS-Erkrankungen – insbesondere die Neuromyelitis optica – sowie eine Kleingefäßvaskulitis wurden ausgeschlossen und stellen in dieser Konstellation wichtige Differenzialdiagnosen dar. Die Therapie erfolgt primär immunsuppressiv. Ob eine zusätzliche antivirale Therapie das Behandlungsergebnis dieser Patienten verbessert, ist aktuell unklar [39]. Bei der Pathogenese der Myelitis scheint aber eine HCV-induzierte Autoimmunität, wie auch bei anderen extrahepatischen Manifestationen, eine besondere Rolle zu spielen [40].

### Enzephalitis

Verschiedene inflammatorische und demyelinisierende Erkrankungen des Hirnparenchyms sind – allesamt insgesamt selten – im Kontext der HCV-Infektion beschrieben. Dabei handelt es sich um Fälle von Patienten mit akuter disseminierter Enzephalomyelitis (ADEM), mit Multiple-Sklerose-ähnlichen Läsionen sowie Enzephalitiden [41]. Die ADEM ist sowohl bei akuter als auch chronischer HCV-Infektion beschrieben [42]. In sehr seltenen Fällen ist das Auftreten fulminanter Enzephalitiden dokumentiert worden [43]. Ein mögliches therapeutisches Ansprechen auf antivirale Therapien ist bislang nicht beschrieben.

- HCV-assoziierte kognitive Defizite stellen eine häufige Komplikation dar und können einen relevanten Einfluss auf die Lebensqualität der Patienten haben.
- Kognitive Defizite bei der HCV-Infektion lassen sich durch eine antivirale Therapie kausal therapieren.
- Myelitiden und demyelinisierende ZNS-Erkrankungen stellen sehr seltene Komplikationen der HCV-Infektion dar.

## Die HCV-Infektion als Risikofaktor

Die aktive HCV-Infektion erhöht das Risiko sowohl für den ischämischen als auch den hämorrhagischen Schlaganfall. Interessanterweise ist das Risiko, einen ischämischen Schlaganfall zu erleiden, auch unabhängig vom Vorliegen anderer kardiovaskulärer Risikofaktoren erhöht [44]. Besonders bei jüngeren Patienten erhöht eine HCV-Infektion das Risiko für intrazerebrale Blutungen bzw. hämorrhagische Schlaganfälle – möglichweise durch chronische vaskulitische Veränderungen der kleinen Gefäße [45]. Eine HCV-Eradikation senkt in dieser Patientengruppe das Risiko für ein erneutes Blutungsereignis [46]. Auch Patienten mit ischämischen Läsionen auf dem Boden einer HCV-assoziierten (in der Regel kryoglobulinämischen) ZNS-Vaskulitis profitieren von einer HCV-Eradikation, zusätzlich zu einer immunmodulierenden Therapie [47].

Ein interessanter Zusammenhang zur HCV-Infektion hat sich auch beim Morbus Parkinson gezeigt. Eine Infektion mit dem Hepatitis-B- oder dem Hepatitis-C-Virus erhöht das Risiko deutlich (RR 1,51–1,76), an einem Morbus Parkinson zu erkranken [48]. Ob dies auf eine direkte Neurotoxizität des HCV, zirkulierende proinflammatorische Zytokine oder andere Faktoren zurückzuführen ist, bleibt aktuell unklar. Allerdings konnten erste Arbeiten zeigen, dass eine erfolgreiche antivirale Therapie das Risiko, an einem Morbus Parkinson zu erkranken, zu senken vermag [49].

## Zusammenfassung

Die HCV-Infektion ist eine im klinischen Alltag häufig anzutreffende Erkrankung, die auch aus neurologischer Sicht relevant ist. Mehr als jeder zweite HCV-infizierte Patient leidet unter neurologischen bzw. neuropsychiatrischen Folgen der Infektion. Dabei stellen Neuropathien und kognitive Defizite die häufigsten Komplikationen dar. Neuropathien können auch ohne Nachweis von Kryoglobulinen auftreten. Die HCV-Infektion sollte zukünftig in die Differenzialdiagnose von (Small-fiber‑)Neuropathien und kognitiven Defiziten ebenso mit einbezogen werden wie in die Abklärung zerebrovaskulärer Ereignisse. Der Nachweis einer HCV-Infektion hat eine hohe therapeutische Konsequenz, denn die chronische Hepatitis C stellt heute in >90 % der Fälle eine kurativ behandelbare Erkrankung und damit einen behandelbaren Risikofaktor dar.
